# Research on medical errors exhibits diverse associations with global indicators of science, development, and health across geographic regions: A scientometrics study

**DOI:** 10.1097/MD.0000000000042985

**Published:** 2025-07-18

**Authors:** Mauro Ali Revolledo Caicedo, Andrés Fernando Montoya Obando, David A. Hernández-Páez, Ornella Fiorillo-Moreno, Johana Galván-Barrios, Patricia Delgado, Ivan David Lozada-Martinez

**Affiliations:** a Universidad de la Costa, Barranquilla, Colombia; b Center for Meta-Research and Scientometrics in Biomedical Sciences, Barranquilla, Colombia; c Clínica Iberoamérica, Barranquilla, Colombia; d Clínica El Carmen, Barranquilla, Colombia; e Biomedical Scientometrics and Evidence-Based Research Unit, Department of Health Sciences, Universidad de la Costa, Barranquilla, Colombia; f Universidad Nacional Autónoma de Nicaragua, Managua, Nicaragua.

**Keywords:** bibliometrics, global health, health care quality indicators, medical errors, meta-research, science

## Abstract

**Background:**

Medical errors are a major public health concern, contributing to increased morbidity, mortality, and healthcare costs worldwide. Understanding the global research landscape on medical errors and its association with key health and development indicators can provide insights into gaps and opportunities for improving patient safety.

**Methods:**

A scientometrics analysis was conducted using data from Scopus, PubMed, and other bibliographic databases. A mixed-methods approach was employed, integrating bibliometric indicators with global health and research metrics. Regression models and meta-analyses were applied to evaluate associations between research productivity and health indicators across 6 World Health Organization regions.

**Results:**

A total of 2639 articles on medical errors were analyzed. The Americas produced the highest volume of research (48.01% of publications) with the highest citation impact (mean 46.5 citations per article). In contrast, Africa had the lowest research output (0.87%). Significant associations were observed between medical error research productivity and health indicators, including reductions in adolescent mortality (Coef. = ‐3676.56, *P* < .001) in the Western Pacific and under-5 mortality (Coef. = ‐0.18, *P* < .001) in the Americas. Each region showed a distinct pattern of associations with the global indicators. Research funding and health expenditure were positively associated with publication output and citation impact.

**Conclusion:**

Medical error research exhibits regional disparities and significant associations with global health indicators. Investment in medical errors research is associated with improved health outcomes in some regions, underscoring the need for equitable resource allocation and enhanced international collaboration to address gaps in patient safety research.

## 1. Introduction

Medical errors represent a significant burden on global health systems, contributing to substantial morbidity, mortality, and economic losses.^[1]^ Estimates suggest that preventable medical errors rank among the leading causes of death worldwide, with millions of adverse events occurring annually.^[2]^ In high-income countries, the financial cost of medical errors is projected to be in the range of billions of dollars due to prolonged hospital stays, additional treatments, and legal claims.^[2]^ In low- and middle-income countries (LMICs), where resources are limited, medical errors exacerbate the existing strain on health systems, reducing overall efficiency and deepening health inequities.^[3]^

Research on medical errors plays a crucial role in identifying patterns, causes, and potential solutions to mitigate their impact.^[4]^ Traditional epidemiological and clinical studies have contributed to understanding medical error mechanisms; however, the translation of such knowledge into policy and practice remains inconsistent across different health systems.^[5]^ One approach to assess the global landscape of research on medical errors is through scientometrics analysis. Scientometrics provides a quantitative evaluation of research output, citation impact, and collaboration patterns, offering insight into how scientific productivity aligns with global health needs and development indicators.^[6]^

A critical aspect of investigating medical errors from a scientometrics perspective is the association between research productivity and global health metrics.^[7]^ Investments in research and development (R&D), current healthcare expenditure (CHE), and socioeconomic determinants of health influence scientific output and the application of research findings in clinical and policy settings.^[8]^ Understanding these relationships is crucial for optimizing research allocation, ensuring that scientific endeavors align with healthcare priorities, and ultimately reducing the burden of medical errors worldwide. The aim of this study was to explore the associations between medical error research and global health and R&D metrics according to geographic regions, providing insights into geographic disparities and the role of scientific research in improving patient safety and health outcomes.

This study was reported following the recommendations of the BIBLIO guideline (Guideline for Reporting Bibliometric Reviews of the Biomedical Literature) and PRISMA (Preferred Reporting Items for Systematic reviews and Meta-Analyses) guideline, which provides standards for reporting scientometrics/bibliometric studies.^[9]^

## 2. Methods

### 2.1. Study design

This was a mixed-methods study.

### 2.2. Data sources

A comprehensive systematic search was performed across multiple academic databases and indexing platforms, including Scopus, PubMed/MEDLINE, the Web of Science Core Collection, SciELO Citation Index, and the KCI-Korean Journal Database. These resources were chosen for their extensive global coverage and substantial repository of bibliographic and citation data in the medical and health sciences. Moreover, their inclusion criteria for peer-reviewed journals adhere to stringent quality standards, making them more robust and reliable compared to alternative sources. The validity and reproducibility of employing these databases for studies of this nature have been previously established.^[10–14]^

### 2.3. Search strategy

A structured search strategy was designed using MeSH terms and their corresponding equivalents to identify peer-reviewed documents that analyze, discuss, investigate, synthesize, or evaluate medical errors within the health sciences. The approach prioritized literature systematically classified within various bibliographic databases, covering disciplines such as medicine, nursing, dentistry, health professions, biochemistry, genetics, molecular biology, immunology, neuroscience, pharmacology, toxicology, and pharmaceutical sciences. In the preliminary phase, pilot tests were conducted by integrating different terms and indexing tags across multiple search engines and databases to refine and optimize the strategy. The final version, which produced the most accurate results when applied in the Scopus database, was as follows: SUBJAREA(HEAL) OR SUBJAREA(DENT) OR SUBJAREA(NURS) OR SUBJAREA(MEDI) OR SUBJAREA(BIOC) OR SUBJAREA(IMMU) OR SUBJAREA(NEUR) OR SUBJAREA(PHAR) AND TITLE(“Medical Errors”) OR TITLE(“Diagnostic Errors”) OR TITLE(“Medication Errors”). This strategy was adapted for use in each of the other databases or search engines.

### 2.4. Time period

The literature search was executed on July 25, 2024, in both English and Spanish. The preliminary screening of titles and abstracts took place between July 28 and September 29, 2024. A subsequent review phase was conducted from September 30 to November 22, 2024, to finalize data extraction related to key scientometrics domains and specific health indicators.

### 2.5. Eligibility criteria

To ensure the relevance and reliability of the data analyzed, studies were included in the analysis only if they met specific inclusion criteria. These were: (A) being peer-reviewed scientific publications indexed in regularly issued academic journals, which guarantees a minimum standard of editorial and scientific quality; (B) availability of the full-text version, which was essential to allow in-depth examination and data extraction; and (C) a clearly defined general objective explicitly focused on the analysis, discussion, investigation, synthesis, or evaluation of mental health.

To preserve the integrity of the dataset, documents were excluded if they: (A) corresponded to publications with non-regular peer review process such as conference proceedings, errata, or retracted articles; (B) lacked essential bibliographic metadata (e.g., author names, journal title, or corresponding author information), which limited traceability and verification; or (C) were still in press at the time of the search and therefore lacked a finalized version suitable for analysis.

Although the primary focus was on English, Portuguese and Spanish literature, documents published in other languages were also eligible, provided they included an abstract in either English, Portuguese or Spanish and met all inclusion criteria while avoiding any exclusion criterion. Finally, considering the historical dimension of this study and its scope, no lower limit was applied to the year of publication, allowing for a longitudinal and comprehensive view of the evolution of the field.

### 2.6. Data standardization

The search results from all databases were exported in .CSV format, preserving all available metadata, including document titles, author names and affiliations, keywords, publication year, citation count, publication type, and other relevant details. Initially, 2 researchers independently conducted a manual review using Rayyan to eliminate duplicate records and assess titles and abstracts to confirm adherence to the inclusion and exclusion criteria. This screening process was performed using Microsoft Office Excel 2016.

Following this, a second independent evaluation was carried out by 2 researchers to complete data extraction related to scientometrics domains, healthcare quality indicators, and global health metrics. In instances of disagreement, a third evaluator was consulted to resolve inconsistencies. Additionally, efforts were made to standardize variables to enhance dataset uniformity. For example, all articles classified as reviews—irrespective of their methodological approach (narrative reviews, systematic reviews with or without meta-analysis)—were consolidated under a single category labeled “reviews.” Similarly, for the variable “country,” the nationality of the corresponding author was used as the reference.

### 2.7. Data synthesis and analysis

To examine the scientometrics domain, data were extracted on the quartile ranking and h-index of each publication, adjusted to the corresponding reference year. These indicators were obtained from the historical records of Scimago Journal & Country Rank (available since 1999) and the Journal Citation Report (available since 1997), with the highest metric for the journal in which the document was published being selected.

Furthermore, countries were categorized into geographic regions using the following classifications: The Americas, Europe, Western Pacific, Eastern Mediterranean, South-East Asia, and Africa (countries/areas by World Health Organization [WHO] regions).^[15]^ Additionally, economic classification was applied based on World Bank criteria, grouping countries into 4 income levels: low-income, LMICs, upper-middle-income, and high-income countries. This classification was based on the most recent data available for 2024.^[16]^

To address domains related to healthcare quality indicators, global health, R&D metrics, quantitative variables directly linked to CHE, disease burden, and R&D activities were used. Twenty-three health indicators—including those related to mortality, disease prevalence, healthcare capacity, access, and expenditure—were obtained, stratified by region and available years. The data were recorded in their original units of measurement. This information was obtained from open-access databases, including those of the World Bank,^[17]^ the WHO’s Global Health Observatory,^[18]^ and the Global Observatory on Health Research and Development.^[19]^

To evaluate the relationships between health indicators and bibliometric metrics, linear regression models were applied. Each health indicator was analyzed against 3 bibliometric metrics (number of publications, total citations, and average H-index), with variables designated as dependent or independent based on the specific analysis. These models were constructed separately for each WHO region to account for potential regional differences. Only significant models with relevant effect sizes are reported; however, all results are available in Supplementary Material S1, Supplemental Digital Content, https://links.lww.com/MD/P293.

Subsequently, we constructed a regression coefficient matrix using normalized *Z*-scores for each bibliometric indicator. This matrix was then subjected to hierarchical clustering based on Euclidean distance and the complete linkage method. To effectively visualize the regression coefficients, heatmaps were generated. The abbreviations of the indicators used can be found in Supplementary Material S2, Supplemental Digital Content, https://links.lww.com/MD/P294.

A meta-analysis was conducted on the regression results. For each health indicator, the coefficients and their standard errors from the regional models were used as inputs for a random-effects meta-analysis. This approach accounted for heterogeneity across regions and provided pooled estimates of the associations. The restricted maximum likelihood method was used to estimate between-region variance. Only significant meta-analysis results are reported; however, all meta-analysis results are available in Supplementary Material S3, Supplemental Digital Content, https://links.lww.com/MD/P295.

All statistical analyses were conducted using R software (version 4.3.1).^[20]^ The scripts for these analyses, along with detailed annotations, are https://doi.org/10.5281/zenodo.14837929.

### 2.8. Ethical statements

This study was approved by the Scientific Committee of Universidad de la Costa (code GRA.2021-07-002-19). However, no humans, animals, or medical records were used as units of analysis.

## 3. Results

### 3.1. Medical errors-related publications: WHO regional trends and characteristics

**Figure 1.**
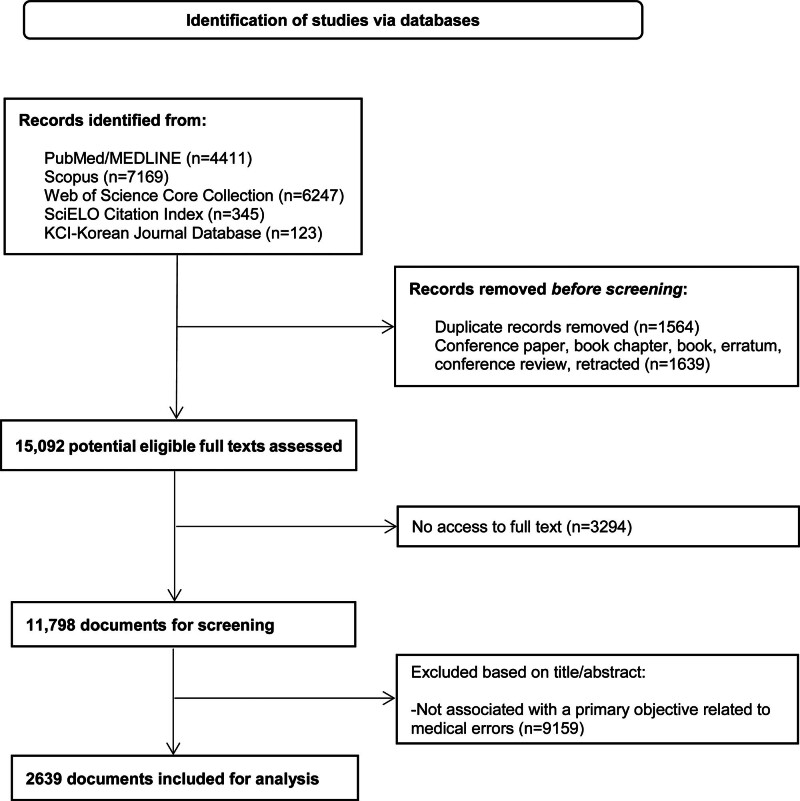
Documents selection flow diagram.

Among the 2639 articles published across the 6 WHO regions (Fig. 1), the Americas accounted for the largest share of publications (48.01%), as well as the highest average citations per paper (46.5) and H-index (115.7). However, it exhibited the lowest proportion of open-access articles (0.44). In contrast, the African, South-East Asian, and Eastern Mediterranean regions had the highest proportions of open-access articles, with ratios of 2.28, 1.19, and 1.01, respectively (Table 1).

**Table 1:** General characteristics of medical errors-related publications by region (N = 2639).

	Americas (N = 1267)	Europe (N = 789)	Western Pacific (N = 269)	Eastern Mediterranean (N = 199)	South-East Asia (N = 92)	Africa (N = 23)
Publications, %	48.01	29.9	10.19	7.54	3.49	0.87
Total citations (mean citations per paper)	58,931 (46.5)	15,951 (20.2)	5110 (18.9)	1883 (9.4)	925 (10)	175 (7.6)
H-index, mean (SD)	115.7 (106.8)	69.2 (108.2)	77.8 (108.6)	47.8 (100.2)	53.6 (99.2)	49.5 (90.2)
Open access/no open access	393/874	313/476	110/159	100/99	50/42	16/7
Document type (N = according to region) (%)						
Article	817 (64.5)	535 (67.8)	210 (78.1)	169 (84.9)	67 (72.8)	22 (95.7)
Editorial	53 (4.2)	28 (3.5)	14 (5.2)	0	3 (3.3)	0
Letter	50 (3.9)	38 (4.8)	6 (2.2)	8 (4)	8 (8.7)	1 (4.3)
Note	47 (3.7)	31 (3.9)	2 (0.7)	2 (1)	1 (1.1)	0
Review	248 (19.6)	140 (17.7)	36 (13.4)	20 (10.1)	12 (13)	0
Short survey	52 (4.1)	17 (2.2)	1 (0.4)	0	1 (1.1)	0
Journal quartile (N = according to region) (%)						
Q1	633 (56.1)	257 (37.4)	123 (49.2)	38 (22.4)	14 (19.4)	5 (23.8)
Q2	284 (25.2)	151 (22)	70 (28)	47 (27.6)	21 (29.2)	6 (28.6)
Q3	142 (12.6)	126 (18.3)	41 (16.4)	58 (34.1)	19 (26.4)	8 (38.1)
Q4	69 (6.1)	153 (22.3)	16 (6.4)	27 (15.9)	18 (25)	2 (9.5)

**Figure 2.**
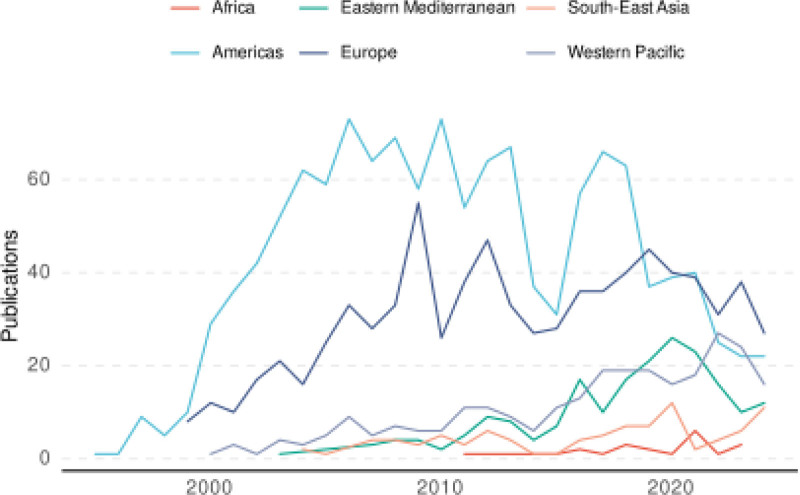
Annual trends in medical errors-related publications by WHO region (1995–2024). Variations in number of publications among the 6 WHO regions over time.

First-publication dates varied significantly between regions, with the Americas recording the earliest publication in 1995, while Africa registered its first publication much later, in 2011 (Fig. 2).

**Figure 3.**
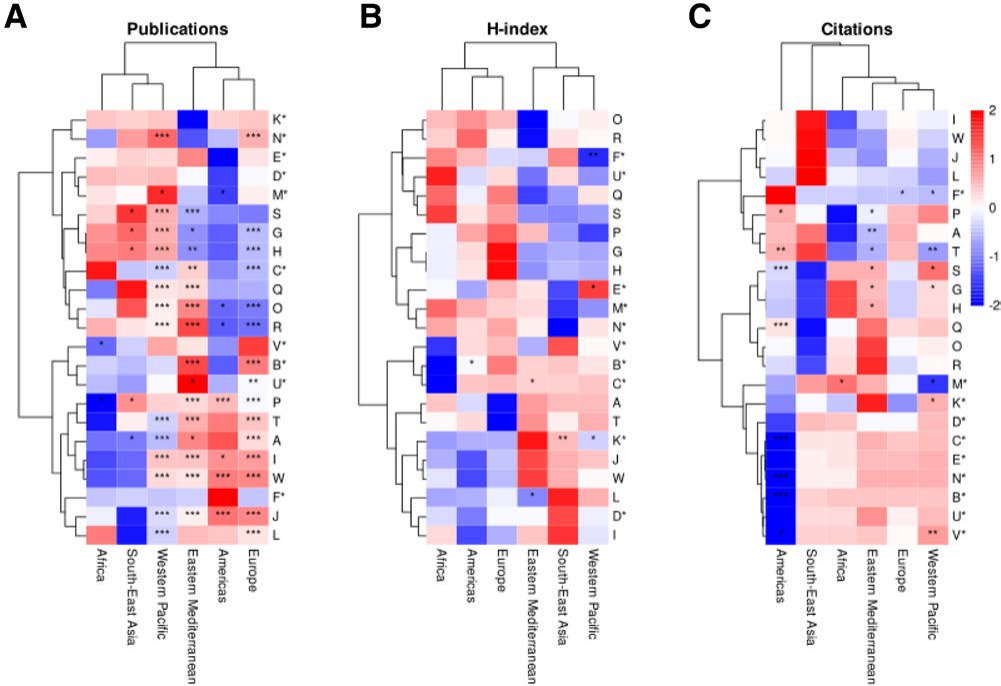
Heatmap of normalized linear regression coefficients (*Z*-scores) for the number of publications (A), average H-index (B), and total citations (C), stratified by WHO region. Indicators are represented by letters, with asterisks denoting those used as independent variables in the models. Statistical significance is indicated as follows: *P < .05, **P < .01, ***P < .001.

More than 60% of publications in each region were categorized as the “Article” document type, followed by “Review” articles. Among articles with a reported quartile ranking (n = 2328), the Americas was the only region where over half of the articles were published in Q1 journals, followed by the Western Pacific (49.2%) and Europe (37.4%). In contrast, the South-East Asian region had the lowest proportion of Q1 publications (19.4%) and the highest proportion of Q4 articles (25%) compared to the other regions. Metrics and publications related to research on medical errors exhibited statistically significant and diverse associations across geographic regions (Fig. 3).

### 3.2. Western Pacific: publications and funding related to reduced adolescent and noncommunicable disease mortality

Analysis in the Western Pacific revealed that each additional publication was associated with a substantial decrease of approximately 3677 deaths among adolescents aged 10 to 19 years (Coef. = ‐3676.56, SE = 551.32, t = ‐6.67, *P* < .001). This model accounted for 67.9% of the variance (*R*² = 0.679) and was statistically significant (*F* = 44.47, *P* < .001). Additionally, increased official development assistance (ODA) for medical research and basic health sectors per capita correlated with more publications (Coef. = 20.17, SE = 7.40, t = 2.73, *P* = .034), explaining 55.3% of the variance (*R*² = 0.553).

Furthermore, a higher number of citations was linked to a lower noncommunicable disease (NCD) mortality rate (Coef. = ‐0.25, SE = 0.084, t = ‐2.91, *P* = .009), with the model explaining 32% of the variance. The Universal Health Coverage Service Coverage subindex positively influenced citations (Coef. = 58.05, SE = 11.48, t = 5.06, *P* = .004), accounting for 83.6% of the variance. Additionally, health-related R&D expenditure as a percentage of total gross domestic expenditure on R&D was positively associated with the H-index (Coef. = 6.40, SE = 2.29, t = 2.79, *P* = .049), explaining 66.1% of the variance. No significant associations were found between the average H-index or citations and mortality indicators.

### 3.3. Europe: health expenditure and publications significantly reduce adolescent mortality

In Europe, each additional publication was associated to a reduction of approximately 1055 adolescent deaths (Coef. = ‐1055.20, SE = 176.61, t = ‐5.97, *P* < .001), with the model explaining 61.9% of the variance (*R*² = 0.619). CHE as a percentage of gross domestic product was positively associated with publications (Coef. = 13.72, SE = 3.15, t = 4.36, *P* < .001), accounting for 47.5% of the variance (*R*² = 0.475).

No significant associations were observed between citations and either mortality or expenditure indicators, nor between the H-index and these variables.

### 3.4. Africa: increased publications reduce hypertension prevalence and enhanced ODA boosts citations

In Africa, an increase in publications was associated with a 0.51% reduction in hypertension prevalence among adults aged 30 to 79 years (Coef. = ‐0.51, SE = 0.21, t = ‐2.46, *P* = .049), explaining 50.3% of the variance (*R*² = 0.503). Additionally, higher ODA per capita correlated with more citations (Coef. = 29.60, SE = 10.52, t = 2.81, *P* = .037), accounting for 61.3% of the variance (*R*² = 0.613).

There were no significant associations between publications and CHE, citations and mortality, or the H-index with mortality or expenditure indicators.

### 3.5. Americas: publications and citations significantly lower under-5 and NCD mortality rates

The Americas demonstrated that each additional publication was associated with a decrease of 0.18 in the under-5 mortality rate (Coef. = ‐0.18, SE = 0.041, t = ‐4.41, *P* < .001), explaining 42.8% of the variance (*R*² = 0.428). Additionally, each citation corresponded to a reduction of approximately 243 NCD deaths (Coef. = ‐242.69, SE = 58.03, t = ‐4.18, *P* < .001), with the model accounting for 49.3% of the variance (*R*² = 0.493).

No significant positive associations were found between publications or citations and expenditure indicators, nor between the H-index and mortality or expenditure.

### 3.6. South-East Asia: publications reduce adult mortality and grants enhance research impact

In South-East Asia, each additional publication was associated with a reduction of 2.61 in the adult mortality rate (Coef. = ‐2.61, SE = 1.10, t = ‐2.38, *P* = .031), explaining 27.5% of the variance (*R*² = 0.275). Moreover, an increase in grants by WHO region and income group was positively associated with the H-index (Coef. = 0.13, SE = 0.029, t = 4.47, *P* = .007), accounting for 80.0% of the variance (*R*² = 0.800).

No significant associations were detected between publications and expenditure, citations with mortality or expenditure, or the H-index with mortality indicators.

### 3.7. Eastern Mediterranean: publications, expenditure, and H-index significantly reduce NCD and adolescent mortality

The Eastern Mediterranean region showed that each additional publication was associated with a decrease of 3.16 in the NCD mortality rate (Coef. = ‐3.16, SE = 0.65, t = ‐4.87, *P* < .001), explaining 66.4% of the variance (*R*² = 0.664). CHE as a percentage of gross domestic product was positively related to publications (Coef. = 17.30, SE = 2.48, t = 6.96, *P* < .001), accounting for 76.4% of the variance (*R*² = 0.764). Additionally, each citation was associated with a reduction of 0.21 in the NCD mortality rate (Coef. = ‐0.21, SE = 0.086, t = ‐2.42, *P* = .032), explaining 32.8% of the variance (*R*² = 0.328). Furthermore, the H-index was negatively associated with adolescent deaths, with each unit increase reducing deaths by approximately 149 (Coef. = ‐149.01, SE = 58.24, t = ‐2.56, *P* = .022), accounting for 30.4% of the variance (*R*² = 0.304).

No significant positive associations were found between citations and expenditure or between the H-index and expenditure indicators.

### 3.8. Medical error publications: meta-analysis of associations with mortality, morbidity, life expectancy, and health expenditure

The meta-analysis revealed significant associations between bibliometric metrics and health indicators across 3 groups: mortality, morbidity and life expectancy, and health expenditure and coverage (Table 2). Among mortality indicators, increased publications were related to reduced mortality rates, including adult mortality (‐1.21; 95% CI: −2, −0.42), neonatal mortality (‐0.29; 95% CI: −0.47, −0.11), under-5 mortality (‐0.61; 95% CI: −0.99, −0.23), children mortality (‐0.04; 95% CI: −0.07, −0.01) and NCD mortality (‐4.44; 95% CI: −7.27, −1.61).

**Table 2:** Significant meta-analysis results of indicators used as dependent and independent variables in linear models related to number of publications.

	Coefficient (95% CI)	Weight
Mortality indicators		
Adult mortality rate (probability of dying between 15 and 60 years per 1000 population)	−1.21 (−2, −0.42)	88.59
Mortality rate among children ages 5 to 9 years (per 1000 children aged 5)	−0.04 (−0.07, −0.01)	216236.7
Neonatal mortality rate 0 to 27 days (per 1000 live births) (SDG 3.2.2)	−0.29 (−0.47, −0.11)	5945.92
Total NCD mortality rate (per 100,000 population) [age-standardized]	−4.44 (−7.27, −1.61)	9.67
Under-5 mortality rate (per 1000 live births) (SDG 3.2.1)	−0.61 (−0.99, −0.23)	1096.51
Morbidity and life expectancy indicators		
Prevalence of diabetes	0.15 (0.05, 0.25)	18599.7
Prevalence of obesity among adults (BMI ≥ 30 [age-standardized estimate])	0.22 (0.11, 0.32)	5499.14
Healthy life expectancy at birth (years)	0.12 (0.04, 0.2)	12856.51
Life expectancy at birth (years)	0.15 (0.05, 0.25)	8419.11
Health expenditure and coverage indicators		
Out-of-pocket expenditure per capita in US$*	0.09 (0.03, 0.15)	6829.67
UHC Service Coverage subindex on noncommunicable diseases*	2.38 (0.2, 4.55)	28.48

NCD = noncommunicable disease, UHC = Universal Health Coverage.

* Used as independent variables in the model.

For morbidity and life expectancy, publication volume was positively associated with the prevalence of diabetes (0.15; 95% CI: 0.05, 0.25) and obesity (0.22; 95% CI: 0.11, 0.32), as well as improvements in healthy life expectancy (0.12; 95% CI: 0.04, 0.2) and life expectancy at birth (0.15; 95% CI: 0.05, 0.25). Regarding health expenditure and coverage, these indicators were used as independent variables to predict publication volume. Higher out-of-pocket healthcare expenditure (0.09; 95% CI: 0.03, 0.15) and improvements in the Universal Health Coverage service coverage subindex for NCD (2.38; 95% CI: 0.42, 4.55) were associated with an increased number of publications.

## 4. Discussion

The findings of this study reveal diverse regional patterns in research productivity, impact, and alignment with global health indicators. The Americas emerged as the most prolific contributor to medical error research, with the highest citation impact and journal quality indicators. Conversely, regions such as Africa and South-East Asia exhibited lower research productivity, with a notable proportion of publications in lower-quartile journals. These disparities underscore the uneven distribution of research efforts, which may be influenced by factors such as funding availability, institutional research capacity, and regional health priorities.^[21,22]^

A significant observation from our study is the correlation between medical error research output and health indicators, such as mortality rates and CHE. In regions with higher investments in healthcare R&D, such as the Western Pacific and Europe, increased research activity was associated with improved health outcomes, including reduced adolescent mortality and NCD burden. These findings suggest that investments in scientific research contribute not only to advancing knowledge but also to tangible improvements in public health.^[23,24]^ However, causality cannot be inferred solely from bibliometric or scientometrics associations,^[25]^ and additional contextual factors, such as health system efficiency and policy implementation, must be considered.

One possible explanation for the observed associations between research activity and health outcomes lies in the role of evidence-based policy development.^[26]^ Countries with well-funded research institutions and strong collaborations between academia and policymakers are more likely to translate research findings into actionable strategies.^[27]^ In contrast, regions with limited research capacity may struggle with implementation gaps, where scientific knowledge does not effectively influence clinical practice or public health policies. This highlights the need for targeted initiatives that bridge the gap between research and policy application, ensuring that medical error research contributes meaningfully to health system improvements.^[28]^

From a policy perspective, our results highlight the need for equitable research funding allocation and international collaboration.^[29]^ Regions with lower research output may benefit from targeted funding initiatives and capacity-building programs aimed at strengthening scientific infrastructure.^[29]^ Moreover, fostering global partnerships can facilitate knowledge transfer and enhance the relevance of medical error research across diverse healthcare settings.^[30]^ The establishment of cross-regional research networks and joint funding mechanisms could help mitigate disparities and ensure that medical error research addresses the most pressing healthcare challenges in all regions.^[30]^

The implications of this study extend beyond scientific output and citation metrics. By demonstrating associations between research productivity and key health indicators, our findings underscore the broader role of scientific inquiry in shaping healthcare outcomes. The observed associations suggest that investments in research do not merely generate academic knowledge but also contribute to the strengthening of health systems, particularly in areas such as patient safety, diagnostic accuracy, and treatment effectiveness. Recognizing this relationship is crucial for global health organizations and funding bodies aiming to maximize the impact of research investments.

Despite the study’s strengths, including its comprehensive data extraction and advanced analytical methods, certain limitations must be acknowledged. The reliance on bibliometric databases may introduce selection bias, as non-indexed research from LMICs may be underrepresented. Additionally, the study design does not allow for causal inferences, necessitating further investigation through mixed-methods research that incorporates qualitative assessments of policy impact. Future studies could explore case-based approaches, examining how specific research initiatives on medical errors have influenced healthcare improvements in different regions.

Future research should focus on examining the implementation of medical error reduction strategies and their translation into clinical practice. Additionally, interdisciplinary approaches integrating artificial intelligence and big data analytics could enhance the precision and applicability of medical error research, leading to more effective interventions.^[31]^ Addressing current gaps in research dissemination and application will be critical to ensuring that medical error research translates into meaningful reductions in adverse events, ultimately improving patient safety on a global scale.

## 5. Conclusions

This study provides a comprehensive assessment of the global research landscape on medical errors and its association with global health, science and development indicators. The findings underscore regional disparities in research output. These disparities highlight broader global inequities in research funding, infrastructure, and scientific collaboration. Increased research activity on medical errors is associated with improvements in global health metrics, particularly reductions in mortality rates and enhanced healthcare system efficiency. Regions with greater investments in R&D showed stronger associations between research output and improved health outcomes, emphasizing the critical role of scientific inquiry in shaping public health policies and clinical practices. Future research should explore the long-term impact of medical error investigations on policy implementation and healthcare quality.

## Author contributions

**Conceptualization:** Mauro Ali Revolledo Caicedo, Andrés Fernando Montoya Obando, David A. Hernández-Páez, Ornella Fiorillo-Moreno, Johana Galván-Barrios, Patricia Delgado, Ivan David Lozada-Martinez.

**Formal analysis:** Mauro Ali Revolledo Caicedo, Andrés Fernando Montoya Obando, David A. Hernández-Páez, Ornella Fiorillo-Moreno, Johana Galván-Barrios, Ivan David Lozada-Martinez.

**Investigation:** Mauro Ali Revolledo Caicedo, Andrés Fernando Montoya Obando, David A. Hernández-Páez, Ornella Fiorillo-Moreno, Johana Galván-Barrios, Patricia Delgado, Ivan David Lozada-Martinez.

**Methodology:** David A. Hernández-Páez, Ornella Fiorillo-Moreno, Johana Galván-Barrios, Ivan David Lozada-Martinez.

**Writing – original draft:** Mauro Ali Revolledo Caicedo, Andrés Fernando Montoya Obando, David A. Hernández-Páez, Ornella Fiorillo-Moreno, Johana Galván-Barrios, Patricia Delgado, Ivan David Lozada-Martinez.

**Writing – review & editing:** Mauro Ali Revolledo Caicedo, Andrés Fernando Montoya Obando, David A. Hernández-Páez, Ornella Fiorillo-Moreno, Johana Galván-Barrios, Patricia Delgado, Ivan David Lozada-Martinez.

## Supplementary Material


