# Supplementary material for: Research on medical errors exhibits diverse associations with global indicators of science, development, and health across geographic regions: A scientometrics study
Source: Medicine (Baltimore). 2025 Jul 18;104(29):e42985. doi: 10.1097/MD.0000000000042985 (PMC12282701; doi:10.1097/MD.0000000000042985)
Supplement: Supplementary file 2 [file medi-104-e42985-s002.docx]

**Appendix**

**Table S1.** Abbreviations of the indicators used.

| **Letter** | **Indicator** |
| --- | --- |
| A | Adult mortality rate (probability of dying between 15 and 60 years per 1000 population) |
| B* | Current health expenditure (CHE) as percentage of gross domestic product (GDP) (%) |
| C* | Domestic general government health expenditure (GGHE-D) per capita in US$ |
| D* | Gross domestic R&D expenditure on health (health GERD) as a % of gross domestic product (GDP) |
| E* | Gross domestic R&D expenditure on health (health GERD) as a % of total GERD |
| F | Health researchers (in full-time equivalent) per million inhabitants, by WHO Region |
| G | Healthy life expectancy (HALE) at birth (years) |
| H | Life expectancy at birth (years) |
| I | Mortality rate among children ages 5 to 9 years (per 1000 children aged 5) |
| J | Neonatal mortality rate (0 to 27 days) per 1000 live births) (SDG 3.2.2) |
| K* | No. of grants by recipient's WHO region and income group |
| L | Number of deaths among adolescents 10-19 years of age |
| M* | Official development assistance (ODA) for medical research and basic health sectors per capita, by recipient country |
| N* | Out-of-pocket expenditure (OOP) per capita in US$ |
| O | Prevalence of diabetes |
| P | Prevalence of hypertension among adults aged 30-79 years |
| Q | Prevalence of insufficient physical activity among adults aged 18+ years (crude estimate) (_) |
| R | Prevalence of obesity among adults, BMI = 30 (age-standardized estimate) () |
| S | Total NCD Deaths (in thousands) |
| T | Total NCD mortality rate (per 100 000 population) , age-standardized |
| U* | UHC Service Coverage sub-index on noncommunicable diseases |
| V* | UHC Service Coverage sub-index on service capacity an access |
| W | Under-five mortality rate (per 1000 live births) (SDG 3.2.1) |
| ** Used as independant variables in the model.* | |
